# A systematic review of mealtime training for caregivers: effectiveness and social validity

**DOI:** 10.3389/frcha.2026.1758507

**Published:** 2026-06-22

**Authors:** Lisa M. Tereshko, Megan Magrauth, Mary Jane Weiss

**Affiliations:** Endicott College, Institute for Applied Behavioral Science, Beverly, MA, USA

**Keywords:** behavioral mealtime interventions, caregiver training, feeding, food selectivity, mealtime challenges

## Abstract

Common problems faced by families and caregivers of children exhibiting mealtime challenges include food refusal, food selectivity, and/or challenging behaviors. To fully address a mealtime challenge, the inclusion of families and caregivers is essential to ensure the generalization of appropriate mealtime behaviors (e.g., acceptance of foods) to actual familial mealtimes. This literature review includes 49 articles that implemented a training procedure to teach behavioral mealtime interventions to parents or caregivers. The researchers analyzed the effectiveness of the interventions across several dimensions with special attention to outcomes and social validity measures. This study discusses recommendations for best practices for mealtime interventions to be used when training parents and caregivers. Furthermore, the authors provide an analysis of limitations in the current literature along with suggestions for future research.

## Introduction

Mealtime challenges are a common problem that families and caregivers of children face. Approximately 72% of individuals diagnosed with a disability and 45% of individuals not diagnosed with a disability have at least one mealtime difficulty (1). Furthermore, the prevalence of the relatively new diagnosis of avoidant/restrictive food intake disorder (ARFID) affects approximately 0.3%–15.5% of children and adolescents and 0.3%–4.1% of adults (2). Because a high percentage of Board-Certified Behavior Analysts (BCBAs®) work with individuals with varying disabilities, these analysts are often faced with families and caregivers requesting support for mealtime challenges (3, 4). Although it is essential that behavior analysts should have the appropriate skill sets to accurately assess mealtime challenges, they must also be able to effectively train parents or caregivers to implement feeding interventions.

Given the complexities of mealtime challenges, behavior analysts require a broad range of skills to successfully and ethically assess and tackle these challenges. Mealtime challenges can range in severity (e.g., food selectivity to food refusal) and type (e.g., liquid refusal and refusal to self-feed). Within the category of food selectivity, the presenting challenges can range in severity; concerns may arise when the individual eats foods centered on the particular feature(s) of the food, the texture of the food, and/or the food group of the food. For example, a child may eat only beige foods such as French fries, chicken nuggets, and chips. However, another example can be given of a child eating only beige foods probably also eating only crunchy beige foods such as potato chips, lentil chips, and saltine crackers. The severity of the concern increases when the number, type, and texture of foods refused increases, and this can lead to food refusal where the individual refuses all foods, leading to potential growth reductions, nutritional deficits, the possible need for a medical professional to insert a feeding tube (5), and in some severe cases, even death (4). For individuals with the most severe feeding challenges, the situation is bleak. Very few specialized centers exist to treat such individuals, and they often have long wait lists. There is also a dire shortage of intensive multidisciplinary feeding programs (6).

These mealtime challenges are further complicated by other issues that an individual with the mealtime challenge may face and by problems experienced by the families and caregivers of the individual. As far as the individual is concerned, they may experience various medical side effects that may include nutritional deficits (7), obesity (8), or chronic constipation and hypertension (9). These individuals may also experience behavioral difficulties such as inappropriate mealtime behaviors (IMBs) (e.g., crying, throwing, and aggression) and problems related to attending and learning (7). For the families and caregivers of these individuals, the challenges include an increased stress level, reduced self-esteem, reduced confidence to care for the individual, and increased healthcare costs (5, 10).

Given the various comorbid diagnoses that may exist, paired with medical and social issues that occur because of the aforementioned challenges, parents and caregivers require specialized training to assist their loved ones during mealtimes and make them deal with their problems successfully. In most cases, families may need to collaborate with behavior analysts, other allied health professionals (e.g., occupational therapists, speech and language pathologists, and physical therapists), and medical professionals (e.g., gastroenterologists, nutritionists, and primary care physicians (5, 11). The presenting problems of individuals are highly idiosyncratic and can include issues in swallowing, chewing, digestion, and motor coordination. An increased understanding of the scope of practice of each of the allied health and medical professions will help families in identifying the required expertise and in collaborating successfully with these professionals (12, 13). However, the contextual variables influencing decisions regarding the assessment of and treatment for mealtime challenges are numerous.

One must consider many factors when selecting interventions to target mealtimes for families to enable the family members or caregivers of individuals to continue the interventions after the treatment team exits the scene. Behavior analysts must carefully consider each option for assessment and intervention and share the plan with the family prior to implementation to ensure that the procedures are socially valid; caregivers must feel that the goals and procedures are acceptable to them. For the individual receiving services, embedding choice into the intervention may increase autonomy over the intervention and enhance its social validity for the client and caregivers (14). The concept of social validity is not new to the science but a defining feature of behavior analysis within the principle *applied* (15). However, researchers do not often assess social validity in behavior analytic research as it relies more heavily on subjective measures. When implementing mealtime interventions, social validity measures are critical to ensure caregiver and client participation as most mealtimes occur outside therapy and school settings.

Two prior reviews examined how often researchers measure social validity across feeding intervention studies and found low rates of social validity measures across parent- or caregiver-implemented feeding interventions (16, 17). Aponte et al. (16) conducted a review of parent training for feeding challenges in children with autism and identified 26 studies. The authors found only three studies that noted that researchers included parent participation in the development of the intervention plan and only seven studies that assessed social validity or parent satisfaction. Similarly, Hodges et al. (17) reviewed studies conducted in the United States that assessed feeding interventions for children with autism that incorporated parent training. The authors identified 12 studies and only nine of those studies measured social validity. Although all studies that measured social validity in these reviews noted that the caregivers scored the interventions with either “high satisfaction” or “acceptability,” more research is needed to determine how researchers assess the social validity of these interventions and caregiver training and with whom researchers are assessing social validity. Furthermore, caregivers may find mealtime interventions highly satisfactory, but less is known about client perspectives and parent training experiences. If researchers and behavior analysts do not provide a technological account of how the social validity of both the intervention and the training was assessed, an assessment that includes input from individuals and their caregivers, one could argue that they do not fully understand the true effectiveness of the intervention.

Family members and caregivers need effective training methods, as the ultimate goal for them is to implement the interventions in the absence of professional help. Behavior analysis has researched many methods to train parents and caregivers in various behavior analytic procedures (18, 19), but there is limited research on best practices for training in the assessment and intervention of mealtime challenges (20). Indeed, while extensive information is available about effective parent training procedures, few extend to the mealtime context.

Research is limited in how to train parents and caregivers; there is even less information available about how to best assess the social validity of the training and selected interventions (16, 17, 21). Many behavior analysts have not had significant training in the assessment of and treatment for feeding challenges (22), which may result in questionable implementation of parent training. The purpose of this review was to extend past literature reviews beyond the autism population and across interventions. Specifically, we examined the current literature that reviewed training parents and caregivers on behavioral mealtime interventions that focused on increasing food consumption, in order to both suggest effective training methods and delineate important gaps in the existing literature as they relate to social validity.

## Methods

### Inclusion criteria

For articles to be reviewed for inclusion, the researchers required the following criteria. First, the articles should have been published in a peer-reviewed journal to ensure a certain level of rigor for review. Second, the articles should have been published before October 2025. The researchers did not use a search start date to allow for a greater scope of the review. Third, the purpose of the articles should be to increase food or liquid consumption through a behavior-analytic intervention and discuss the training of the parent or caregiver. Fourth, the authors of the articles should have implemented an experimental design that demonstrated experimental control of the independent variable on the dependent variable (either across the training or feeding behavior). Finally, the articles had to be available in English. If any, or all, of these inclusion criteria were not met, the researchers excluded the articles.

### Search procedure

The authors conducted a systematic literature review on increasing food or liquid consumption of individuals with food selectivity or food refusal following training of a feeder (e.g., parent and caregiver). The researchers conducted the review in accordance with the PRISMA guidelines (23), which stipulate three tiers of review: (1) identification of articles, (2) screening of articles, and (3) inclusion of articles into the review.

The primary researcher conducted the initial searches on four databases: EBSCO (PyscINFO and ERIC), PubMed, ProQuest, and Sage Premier. A doctoral-level student of behavior analysis with a background in behavioral mealtime interventions replicated the searches. All searches were completed in October 2025. The search terms included the following: (feeding OR food selectivity OR food refusal OR picky eating OR mealtime OR Pediatric Feeding Disorder OR Avoidant/Restrictive Food Intake Disorder OR ARFID) AND (behavioral OR ABA OR behavior analysis OR BCBA® OR behavior analyst) AND (training OR supervision OR development OR parent training OR caregiver training).

### Coding procedures

After the articles met the inclusion criteria, the researchers coded all the articles across a variety of measures: (1) trainee demographics, (2) feeding participant demographics, (4) training package, (5) mealtime intervention, (3) dependent variable(s), (6) experimental design, (7) outcomes, (8) generalization and maintenance, and (9) social validity. Across all articles, the researchers recorded data for each measure as defined below. These data can be found within the Supplementary information and summarized across Tables 1–6.

**Table 1:** Training participant demographics.

Characteristic	*n*	%
Age (in years)		
30–34.9	5	33.3^a^
35–39.9	6	40.0^a^
40–44.9	4	26.7^a^
45 plus	1	6.7^a^
Not reported	40	83.3
Role		
Parents	48	98.0
Grandparents	5	10.2
Staff	4	8.2
Behavior analysts	2	4.1
Teacher	1	2.0
Unspecified caregiver	1	2.0
Education		
Vocational/trade school	2	4.1
High school	6	12.2
Associate's	1	2.0
Bachelor's	7	14.3
Master's	4	8.2
Doctoral	1	2.0
Unspecified higher education	6	12.2
Not reported	37	75.5

a Percentages out of only those studies that reported.

Characteristics may not equal 100% as multiple characteristics may have been noted in one study (e.g., parent and staff).

**Table 2:** Feeding participant demographics.

Characteristic	*n*	%
Feeding participant		
Age (in years)		
0–4.9	24	52.2^a^
5–12.9	21	45.7^a^
13–17	1	2.2^a^
Not reported	4	8.2
Race/ethnicity		
White	12	24.5
Asian	7	14.3
Non-Hispanic	5	10.2
Australian	3	6.1
African American	2	4.1
European	2	4.1
Hispanic	2	4.1
New Zealand	2	4.1
Unspecified multiple races	2	4.1
Black	1	2.0
Brazilian	1	2.0
Canadian	1	2.0
Chinese	1	2.0
Filipino	1	2.0
Latino	1	2.0
Middle eastern	1	2.0
Māori	1	2.0
Pacific	1	2.0
South American	1	2.0
Not reported	28	57.1
Diagnosis		
ASD/PDD	30	61.2
Medically based iagnosis	16	32.7
Developmental or intellectual delay	8	16.3
Feeding disorder	8	16.3
Typically developing	8	16.3
Not reported	6	12.2
Feeding concern		
Food selectivity	29	59.2
Food refusal	4	8.2
Varied	8	16.3
Other	2	4.1
Not reported	3	6.1

a Percentages out of only those studies that reported.

Characteristics may not equal 100% as multiple characteristics may have been noted in one study (e.g., White and Non-Hispanic).

**Table 3:** Components of training and interventions.

Component	*n*	%
Training components		
Instructions		
Verbal	26	53.1
Written	23	47.0
Unspecified	8	16.3
Feedback	31	63.3
Modeling	27	55.1
Role-play	15	30.6
Rehearsal	12	24.5
Coaching	9	18.4
Observations	7	14.3
Question and answer sessions	7	14.3
Telehealth	4	8.2
Other	16	32.7
Interventions trained		
Positive reinforcement	38	77.6
Escape extinction	11	22.4
Prompting	9	18.4
Fading	8	16.3
Non-removal of the spoon	8	16.3
Antecedent manipulations	7	14.3
Timeout	6	12.2
Shaping	5	10.2
Response cost	3	6.1
Compliance training	3	6.1
Sequential (high-p/low-p)	3	6.1
Attention extinction	2	4.1
General behavioral guidelines	11	22.4
Other	2	4.1
Dependent variables		
Consumption	44	89.8
Fidelity	30	61.2
Inappropriate mealtime behaviors	34	69.4

Components may not equal 100% as multiple components may have been noted in one study (e.g., rehearsal, modeling, and feedback).

**Table 4:** Experimental designs.

Component	*n*	%
Experimental design		
Multiple baseline/multiple probe	24	49.0
Group	10	20.4
Reversal	8	16.3
Changing criterion	5	10.2
Combined	2	4.1
Other	4	8.2
Fidelity directly measured	30	61.2
With experimental control	9	18.4
Without experimental control	21	42.9

Designs may not equal 100% as multiple designs may have been noted in one study (e.g., multiple baseline and reversals).

**Table 5:** Generalization and maintenance measures.

Component	*n*	%
Generalization		
Intervention	20	40.8
Training	0	0.0
Both	1	2.0
None	28	57.1
Maintenance		
1–4 weeks	6	12.2
5–12 weeks	14	28.6
13–24 weeks	3	6.1
6 months—1 year	5	10.2
Over a year	3	6.1
None	16	32.7

**Table 6:** Social validity measures.

Social validity component	*n*	%
Participant		
Parent	29	59.2
Feeding participant	0	0.0
Teacher	1	2.0
None	19	38.8
Assessment		
Likert scale questionnaire	16	32.7
Formalized assessment	7	14.3
General questionnaire	3	6.1
Modified published assessment	2	4.1
Informally assessed	2	4.1
None	18	36.7
Results		
Mealtime intervention satisfaction	25	80.6^a^
Positive satisfaction	24	96.0^a^
Neutral satisfaction	1	4.0^a^
Training satisfaction	16	51.6^a^
Positive satisfaction	14	87.5^a^
Neutral satisfaction	2	12.5^a^

a Percentages out of only those studies that reported.

Characteristics may not equal 100% as multiple characteristics may have been noted in one study (e.g., White and Non-Hispanic).

#### Trainee demographics

A trainee participant within this review is defined as a person receiving the training to implement the feeding intervention with another individual. Researchers collected information on the number of trainees, the age of the trainees, what role the trainee had toward the feeding participant, and the level of education of the trainee. If the author(s) of an article did not report any of the aforementioned information in their article, the researcher scored the article as “not reported.” The researchers counted participants in both the control and test conditions within the total number of participants. In articles that saw attrition of participants, the researchers recorded the number of participants who completed the article. The researchers categorized articles that reported a mother or father as a “parent” and a grandmother or grandfather as a “grandparent.” If an article reported additional staff or professional trainees, the researchers coded them as “staff” for paraprofessionals, therapists, and direct staff and coded teachers and BCBAs® as such.

#### Feeding participant demographics

A feeding participant in this review is any individual who is receiving the mealtime feeding intervention. Researchers collected data on the number of feeding participants in each article, the age(s) of the feeding participant(s), the race or ethnicity of the participants, the diagnosis, and the level of feeding severity. If the study was a group study, participants in both the control and test conditions were counted within the total number of participants. Based on information provided in each study, the researchers coded age either as the individual age of each participant or as a range and mean. The researchers also recorded the stated race or ethnicity and diagnosis of each feeding participant as noted by the author of each study. Finally, the researchers scored the reported feeding concern by noting whether the participant displayed food selectivity or food refusal or coded “varied” if the author(s) noted both concerns among the participants in the study.

#### Training package

The training package measure assessed the components of the training. The researchers categorized the training components into written and/or verbal instruction (this included didactic training), observations of mealtimes, modeling, role modeling, rehearsal, feedback (e.g., praise, corrective feedback, and performance feedback), coaching (i.e., *in-vivo* prompts and feedback and direction to trainee), question and answer sessions (Q&As), and trainee self-monitoring.

#### Mealtime intervention

Researchers scored all components of the intervention that was trained during the study. If the author(s) implemented and provided a named package in the study, the researchers recorded the named package and the components of the package, if provided.

#### Dependent variable(s)

The researchers scored and categorized each dependent variable of the studies as consumption, IMBs, trainee treatment fidelity, child-feeding behavior, and caregiver stress. Across studies, the author(s) used different terminologies to describe the feeding participant, such as “opening their mouth and swallowing the presented food or liquid,” but the researchers classified all of these as “consumption.” Studies that used indirect assessments measured various child feeding behaviors, caregiver stress, and IMB. If the indirect measure(s) asked for information about the child's consumption, the researchers scored it as “child-feeding behaviors.” If the indirect measure(s) asked for details about the caregivers’ levels of stress due to their perception of the challenge, the researchers scored it as “caregiver stress.” If the indirect measure(s) asked about IMBs during mealtimes, the researchers scored it as IMB.

#### Experimental design

The researchers scored and classified each study based on the experimental design used. If more than one design was combined with another in a study, the researchers recorded all used designs. Furthermore, if the authors measured caregiver fidelity to implement the intervention, the researchers scored if an experimental control was used to assess fidelity.

#### Outcomes

The researchers scored both the outcome of the training program and the outcome of the feeding participant. They scored the outcomes as “improved” if an improvement was noted from baseline levels to the terminal session of intervention or if a percentage of change was reported in the desired direction. If the participants’ response was variable across conditions with high levels of overlap between the intervention and the baseline or if the intervention worked for some participants but not for others, the researchers scored the intervention as “having a varied outcome.”If the studies did not report on the outcome of the training or intervention, the researchers marked it “as not reported.”

#### Generalization and maintenance

The researchers scored each article based on whether the authors assessed generalization of the intervention and/or the training. If the training or intervention was noted to be assessed across a novel dimension (e.g., setting), the researchers scored it for generalization. Then, they scored each article that assessed generalization based on what aspect (i.e., training or intervention) occurred. If an article assessed for generalization of the training and intervention, the researchers marked it as “both.” If neither was scored, the researchers marked it as “no.” The researchers also scored if maintenance was assessed by the author(s) of each study. If the study noted a follow-up data measure after the conclusion of the study, the researchers scored that “the author(s) assessed the maintenance.” If maintenance was assessed, the researchers scored the duration(s) between the last intervention session and the maintenance session(s).

#### Social validity

The researchers scored each article based on the assessment of social validity. If an article contained a social validity measure, the researchers recorded the source of the social validity (i.e., who provided the information). For example, the researchers may have obtained information from the trainee/caregiver. The researchers also scored the type of assessment used to assess social validity. The types included author-generated Likert scale questionnaires or questionnaires, formalized published assessments, modified formal assessments, and informal assessments (e.g., conversations with the participants or parents). The researchers also recorded the outcome of the social validity by recording which aspect was assessed (i.e., training or intervention) and if the results were positive, negative, or neutral. The researchers scored “positive” if the author(s) noted the results as acceptable or satisfied or recorded a score of 4 or higher on a 5-point Likert scale where a score of 1 denoted “strongly disapprove” and 5 denoted “strongly approve.” The researchers scored “negative” if the author(s) noted the results as unacceptable or dissatisfied or recorded a score of 2 or lower on the same 5-point Likert scale. Finally, the researchers scored “neutral” if the authors noted neutral responses across participants toward their acceptance of the intervention or training or recorded a score of 3 on the Likert scale.

### Analysis of rigor: interrater agreement

The analysis of rigor included in this study was both an interrater agreement and interobserver agreement. Although there are additional methods that could be further implemented, the researchers found the present methods appropriate for the current review. To implement an interrater agreement on inclusion of studies across the searches, a doctoral student in behavior analysis completed an independent search using the same databases, keywords, and inclusion criteria. The raters scored an agreement when both raters scored the articles as included or excluded for the review. To calculate the interrater agreement, the researcher took the number of agreements (included or excluded) and divided them by the total number of articles screened and multiplied by 100. The interrater agreement on this measure was 99% (range: 91.7%–99.8%). The primary researcher reevaluated the articles with disagreements to determine eligibility for inclusion. The primary researcher then shared the reason for exclusion or inclusion with the secondary rater and agreement was met. To assess the risk of bias during scoring the measures across articles, a graduate student and the same doctoral student in behavior analysis coded 84% of the measures scored. Their findings were then compared with the primary researcher's data. The interrater agreement was calculated by the number of agreements (data gathered from studies) divided by the total number of articles measured and multiplied by 100. The interrater agreement on this measure was 94% (range: 89.1%–100%). The primary researcher reassessed each measure marked as a disagreement and met with secondary raters to determine accurate collection of data across measures.

## Results

### Number of articles

**Figure 1:**
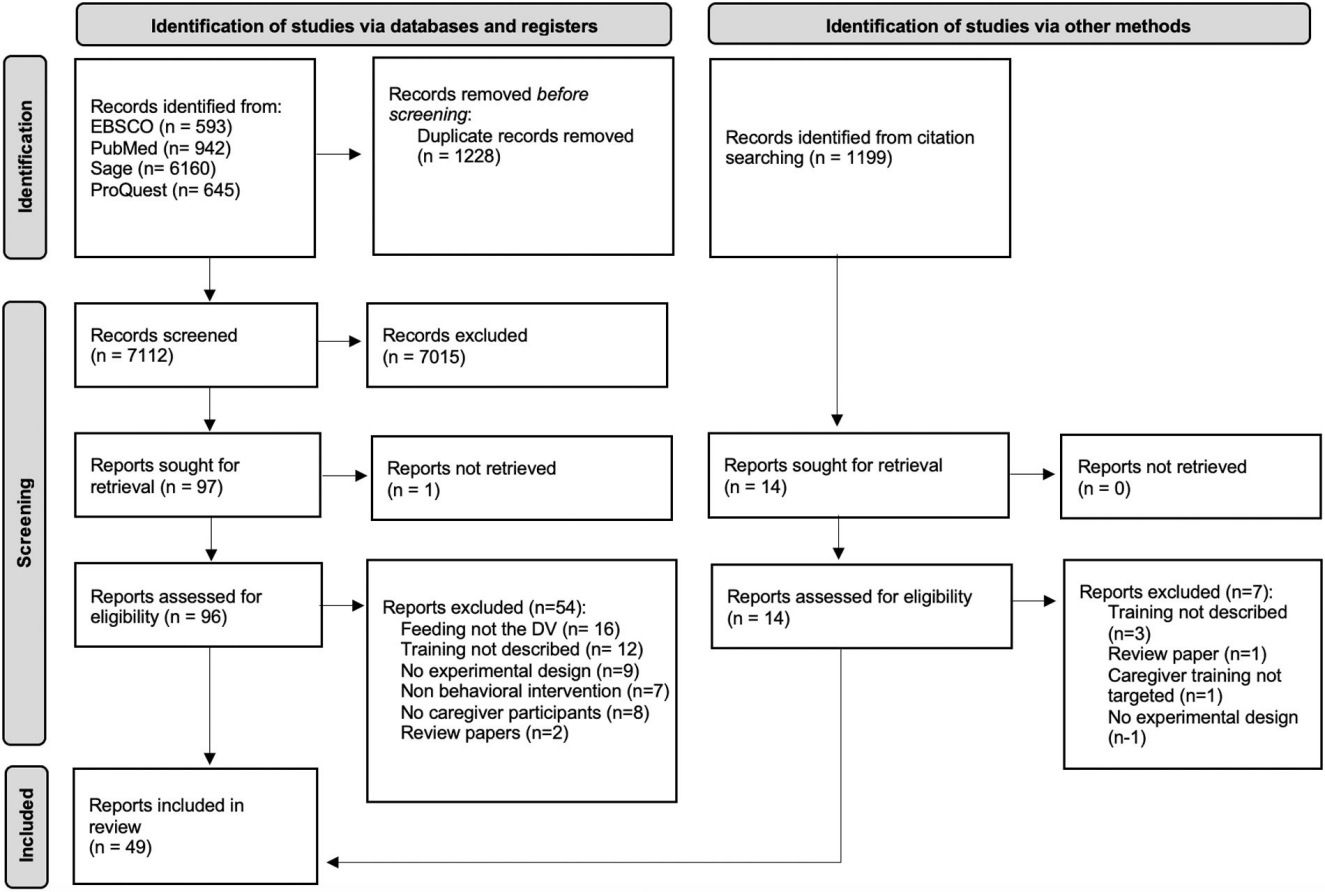
The PRISMA diagram of the search procedure.

The researchers pulled out a total of 8,340 records from the four initial database searches. Of these articles, 1,228 were duplicates, which left 7,112 records for the researchers to screen. After screening the records, the researchers removed 7,015 records, as they did not meet all the inclusion criteria, and sought 97 reports for retrieval. Upon retrieval of the reports (the researchers were unable to retrieve one), the researchers excluded 54 reports, with the majority of reports excluded because of not having “increased feeding” as the dependent variable (*n* = 16). The removal of these reports left 42 reports to be included. Then, the researchers conducted a secondary search by reviewing the references of the 42 included reports, which identified 1199 more records for the researchers to screen. After the additional records were screened, it was found that 14 met the inclusion criteria, following which they were sought for retrieval. Following a review of the full text, seven met the inclusion criteria. The researchers excluded three reports, as the author(s) did not describe the training procedure, two did not target caregiver training, one was a review paper, and one did not have an experimental design. After the initial database searches and the secondary search, 49 articles met the inclusion criteria. See Figure 1 for a full explanation of the search procedure as applied to the PRISMA diagram (23).

### Trainee demographics

A total of 503 trainees participated in 49 studies. Three studies did not report the exact number of trainees as they used general terms such as parents or caregivers, which qualified them for inclusion in the review. There was a mean of 10.9 participants per study (range: 1−124). Forty-eight studies (98.0%) reported training of parent(s) and five studies (10.2%) reported training of grandparent(s). Forty studies (83.3%) did not report the ages of the caregiver participants or reported only an age range, thus removing the ability to determine the mean age of participants. Of the studies that reported individual ages (*n* = 15) or the mean age of participants, five studies (33.3%) reported a mean age of 30−34.9 years old, six studies (40%) a mean age of 35−39.9 years old, four studies (26.7%) a mean age of 40−45.9 years old, and one study (6.7%) reported a mean age of above 46 years old. Thirty-seven studies (75.5%) did not report the education level of the caregiver articles. The most common education levels reported were participants with a bachelor's degree education (*n* = 7, 14.3%) and participants with a high school education (*n* = 6, 12.2%). See Table 1 for further details on trainee participant demographics.

### Feeding participant demographics

A total of 529 feeding participants partook in 49 studies. There was a mean of 10.8 participants per study (range: 1−124). Four studies (8.2%) did not report the ages of the participants or reported only an age range, removing the ability to determine the mean age of participants. Of the studies that reported individual ages (*n* = 46) or the mean age of participants, 24 studies (52.2%) reported a mean age of 0−4 years old, 21 studies (45.7%) a mean age of 5−12 years old, and one study (2.2%) reported a mean age of 13−17 years old. Many ethnicities were reported across the studies; however, 28 studies (57.1%) did not report participants’ ethnicity. The most prevalent ethnicities were White, as noted in 12 studies (24.5%), and Asian, as noted in seven studies (14.3%). All other reported ethnicities can be found in Table 2. Many diagnoses were reported across all participants, with the most common being autism spectrum disorder (ASD) or pervasive developmental disorder (PDD) prevalent in 30 studies (61.2%). Sixteen studies (32.7%) reported a medically based diagnosis, eight studies (16.3%) a typically developing feeding participant, eight studies (16.3%) ARFID or another diagnosed feeding disorder, eight studies (16.3%) reported a developmental or intellectual delay diagnosis, and six studies (12.2%) did not report anything. Furthermore, the feeding concern varied across studies, with 29 studies (59.2%) reporting participants with food selectivity, four studies (8.2%) reporting participants with food refusal, and eight studies (16.3%) reporting participants with both classifications of feeding concerns. Three studies (6.1%) did not specify the feeding concern and two studies (4.1%) used other classifications. See Table 2 for further details on all feeding participant demographics.

### Training package

The training components varied across the studies. Most studies (*n* = 46, 93.9%) combined more than one training component with another component for their training. The most common training component was instruction that was reported in 41 studies (83.7%), with written instruction presented in 23 studies (47.0%), verbal instructions in 26 studies (53.1%), and an unspecified modality of instruction in eight studies (16.3%). Feedback was the next most common component reported in 31 studies (63.3%). Modeling was part of training in 27 studies (55.1%) and role-playing was part of training in 15 studies (30.6%). More information on training packages can be found in Table 3.

### Mealtime intervention

One study (2.0%) used a single intervention, while the remaining studies (*n* = 48; 98.0%) combined interventions for the intervention packages. The most common intervention component was positive reinforcement, which author(s) implemented in 38 studies (77.6%). The next most reported interventions were escape extinction in 11 studies (22.4%) and prompting in nine studies (18.4%). More information on the interventions implemented can be found in Table 3.

### Dependent variable(s)

The researchers scored each study based on the dependent variables measured. Thirty studies (61.2%) directly measured the caregiver training by assessing treatment fidelity following training. The most common dependent variable measured was food or liquid consumption, which the author(s) measured in 44 studies (89.8%), and 34 studies (69.4%) measured inappropriate mealtime behaviors emitted by the feeding participant. More information on the dependent variables measured can be found in Table 3.

### Experimental design

The studies used a variety of experimental designs. Two studies (4.1%) combined two or more experimental designs. The author(s) implemented multiple baseline designs (or multiple probe) most often as scored in 24 studies (49.0%), followed by group designs in 10 studies (20.4%), and reversal designs in eight studies (16.3%). Of the 30 studies that measured caregiver fidelity, only nine studies (30.0%) demonstrated experimental control of the caregiver training and its direct effects on fidelity. More information on the experimental designs and methods of measurement can be found in Table 4.

### Outcomes

The researchers measured outcomes for both the trainee and the feeding participant. The performance of the training participants improved following training reported in 39 studies (79.6%) and showed variable progress in one study (2.0%); the author(s) did not report outcomes in nine studies (18.4%). All articles reported the outcome of the feeding participant. The feeding participant behavior showed improvement in 47 studies (95.9%) and variable progress in two studies (4.1%).

### Generalization and maintenance

The researchers measured whether the studies’ authors assessed generalization of the intervention and training. Twenty-eight studies (57.1%) did not assess generalization. Twenty studies (40.8%) assessed generalization of the intervention, no studies assessed only generalization of the training, and one study (2.0%) assessed generalization of both the intervention and the training. More information on generalization for each study can be found in Table 5.

The researchers also scored if the studies assessed maintenance. Thirty-four studies (69.4%) assessed maintenance of the mealtime behavior and 15 studies (30.6%) did not. Of the studies that assessed maintenance, the duration of time that passed before assessing maintenance ranged from 6 days to 3 years. More information on maintenance assessments for each study can be found in Table 5.

### Social validity

Thirty-one studies (63.2%) assessed social validity, and 18 studies did not (36.7%). Of the studies that assessed social validity (*n* = 31), 30 (96.8%) specified that the authors assessed this component only with the parent(s). One study (3.2%) assessed social validity using teachers and parents as trainees (24). No studies assessed this component with the feeding participants. The author(s) assessed social validity in a variety of ways. In those studies that measured social validity, the most common method used was Likert scale-based questionnaires, which were employed in 16 studies (51.6%), followed by formalized social validity assessments in seven studies (22.6%). Furthermore, of the studies that assessed this component (*n* = 31), 25 studies (80.6%) assessed the social validity of the mealtime intervention, and 24 studies (96.0%) found positive acceptability and satisfaction, with one study (4.0%) reporting neutral findings. Sixteen studies (51.6%) assessed the social validity of the training, and 14 studies (87.5%) found positive acceptability and satisfaction, with two studies (12.5%) reporting neutral findings. More information on the social validity assessments in each study can be found in Table 6.

## Discussion

Mealtime challenges such as food selectivity and refusal are significant issues. The presentation of mealtime challenges often co-occurs with other diagnoses, leading to the need for expert intervention. Caregivers require training in nuanced skills to address these issues. This literature review focused on the evaluation of the literature on training parents and caregivers to implement behavioral mealtime protocols. Information from the literature was summarized across the participants’ demographics, procedural components, outcomes, and indices of quality.

Several factors complicate the issues involved in this realm of clinical service delivery. The young age of the (majority of) feeding participants increases concerns about their vulnerability and underscores the need to train families and caregivers to implement compassionate feeding interventions. Indeed, feeding interventions often focus on younger participants, partly because of the basic need to consume food to sustain life. Errors in implementation strategies can worsen health outcomes and the familial quality of life.

This literature review further supports the need for interventions for younger individuals as most studies (51.1%) included 0−4-year–old participants. Furthermore, most studies within this review reported a participant with ASD or PDD (60.4%), suggesting that the diagnosis may have recently occurred (25). This may increase the potential stress of caregivers (26, 27); behavior analysts must take care to ensure that they support families and that the families are comfortable with the procedures implemented. The frequent use of positive reinforcement as an intervention, or intervention component, may aid in the reduction of caregiver stress, because research has found that parents and caregivers of individuals with autism perceive the term *positive reinforcement* (28) and the procedures (29) implemented in a positive light. However, it is essential to assess the preference of each intervention component with the client and caregivers prior to implementation to ensure assent, consent, and social validity.

For any treatment to be successful and for the studies to demonstrate this effect, there must be strong experimental control and a high level of treatment integrity (30). The researchers noted the experimental designs across all studies; however, they did not examine the strength of the effect across all studies, as most of the data provided within the studies focused on feeding behavior rather than directly on training. Calculating the strength of effect of the training would add value to future studies (e.g., non-overlapping data points). Most studies within this review (62.5%) directly measured parents’ and caregivers’ treatment integrity following training, while the others focused on the outcomes of the intervention to determine the effectiveness of the training. However, without the measure of treatment integrity to ensure exactly which procedures parents and caregivers implemented, the effectiveness of the interventions and trainings could be reduced.

One well-established training technique to teach parents and caregivers performance-based skills is behavior skills training (BST; 31), which incorporates the components of instruction, modeling, rehearsal (or role play), and feedback. Although only 12 studies (25%) within this review implemented all components of BST, the great majority of studies (93.8%) used a combination of training components, with the most common components aligning with BST. A further examination of which component (or a combination of components) is necessary for training parents and caregivers to implement mealtime interventions is required to ensure effective and efficient training.

A noted limitation in the currently available literature is the need for more data on generalization and maintenance. The majority of studies (*n* = 39, 79.6%) included within this review were single-case designs, which may limit the generality of the findings. Researchers need to conduct their studies with a larger participant pool to increase the generalizability of the data gathered. Furthermore, more than half of the studies (58.3%) included in this review did not include any assessment of generalization. Generalization is a key indicator of success of an intervention and of the quality of research studies; it is especially compelling for mealtime interventions, given the necessity for treatment outcomes to generalize to real-life settings. A majority of the studies in this review examined the generalization of the intervention more than the training of the caregiver or parent. However, generalization of the caregiver or parent training is just as essential to ensure their ability to assist their child in novel situations. Information on maintenance was stronger, with most studies (68.8%) assessing the effects of the intervention over time, but more information is needed for a comprehensive understanding of the maintained effects of intervention, of the need for booster sessions, and of long-term outcomes.

As stated previously, the concept of social validity is not new to the science of behavior analysis but is limited within the field's research. This finding was further supported within this review. Over a third of the studies (37.5%) did not assess for social validity of the intervention or training within their study and no studies obtained social validity measures from the feeding participants themselves. Given the focus in the field on assent and on assent-based interventions, this is a glaring omission (19). The types of assessment of social validity and the responses they targeted are also of concern. Of the studies in this review that reported social validity results, the researchers primarily examined the levels of acceptability of the interventions and satisfaction over the results. This is a significant concern; behavior analysts and researchers should also assess the subjective experience of participants; it is important to know whether the training experience was positive for the caregivers and whether the intervention was acceptable for the caregivers and the feeding participants. Behavior analysts and researchers need to assess social validity before, during, and following an intervention to ensure that assessment extends to goals, procedures, and outcomes. Arguably, interventions that caregivers and parents do not view positively will not be implemented with fidelity (32), thus reducing their effectiveness. More information is needed about social validity, especially about the levels of acceptability by clients and caregivers, the feasibility of implementation, and the likelihood of adoption across settings and over time.

From the extant literature, feeding interventions are largely successful and caregiver training helps in ensuring the effective implementation of interventions across settings. Like the trainings used within this review, most of the interventions are multicomponent ones and rely on the use of multiple elements, including positive reinforcement, shaping, and escape extinction. A deeper analysis of the components of intervention is required, and comparative studies would help in elucidating the most effective methods/approaches. It may also be the case that participant profiles are best matched to particular procedural variations, but given the limited demographic information that the author(s) provided on the training participants and the feeding participants, this may be hard to identify at this time. Future research should examine these questions. In addition, future research should incorporate more elaborate assessments of maintenance, generalization, and social validity while providing increased information on participants’ demographics.

With regard to caregiver training, there is a dearth of information about the effective elements of training. It does appear that the elements of training associated with BST are effective in training caregivers in this context. A more nuanced evaluation of the most important elements of training is warranted. In addition, information about the outcomes of training should include more assessments of the maintenance and generalization of training effects, especially given the need for these skills to be demonstrated over time and across mealtime contexts. Finally, more elaborate assessments of social validity could help mitigate issues with long-term adherence and provide caregivers with interventions that they deem acceptable and effective.

Overall, this literature review confirms the need for more research in training parents and caregivers to implement mealtime interventions with an increased attention to the analysis of the rigor within the research. More research is needed on the array of available interventions and how to train each component of the intervention, on the comparative efficacies of caregiver training components and intervention components, and on matching interventions to client profiles. Additional assessments of bias should also be undertaken, with future research addressing caregiver training of mealtime interventions as further assessments are recommended by the PRISMA guidelines, and given the fact that only an interrater agreement was implemented in the current study to address bias. Generalization and long-term maintenance data for caregiver training and mealtime interventions are needed to understand the robustness of the outcomes. Social validity measures are vital to understanding the broader issues associated with real-world implementation. Information from the direct service recipient is also needed, especially to ascertain individuals’ assent and perceptions of the procedures. Future research on parent and caregiver stress levels before and following training and intervention would benefit the field to help in answering the questions surrounding parent and caregiver stress during mealtimes.

This review underscores the strength of behavioral interventions in addressing this challenging context for the caregivers of those with feeding challenges as well as for individuals themselves. Feeding challenges are complex and require nuanced assessment, individualized treatment, and diligent attention to social validity. In training caregivers to support individuals with these challenges, clinicians must ensure that they are competent to conduct the training; such training must be thorough, individualized, based on available evidence, and focused on outcomes that include effectiveness of the procedures, generalization to natural contexts, and comfort with the intervention.

## Data Availability

The original contributions presented in the study are included in the article/Supplementary Material, and further inquiries can be directed to the corresponding author.
