# Supplementary material for: A systematic review of mealtime training for caregivers: effectiveness and social validity
Source: Front Child Adolesc Psychiatry. 2026 Jun 22;5:1758507. doi: 10.3389/frcha.2026.1758507 (PMC13333687; doi:10.3389/frcha.2026.1758507)
Supplement: Supplementary file 1 [file Table1.docx]

**Table 1**

*Participant demographics*

|  | Trainee Participant | | | |  |  | | | | Feeding Participant | | | |  |  |
| --- | --- | --- | --- | --- | --- | --- | --- | --- | --- | --- | --- | --- | --- | --- | --- |
| Article | Number | Role | Age | Education |  | Number | Age(s) | Race | | Diagnosis | | Feeding Severity | | |  |
| Aclan & Taylor, 2017 | 2 | Parents | 50; 36 | Not Reported |  | 2 | 8, 4 | | Not Reported | | ASD | | Not reported | | |
| Alaimo et al., 2018 | 3 | Parents | 32; 34; 27 | Not Reported |  | 3 | 2, 4, 7 | | Not Reported | | TD; PDD; esophageal eosinophilia, lymph nodular, hyperplasia of the colon, food allergies, DD, GER, FTT | | Food selectivity | | |
| Alaimo et al., 2023 | 3 | Parents | 36; 42; 44 | Not Reported |  | 3 | 13, 5, 15 | | Not Reported | | TD; ASD; ASD | | Varied | | |
| Anderson & McMillan, 2001 | 2 | Parents | Not Reported | Not Reported |  | 1 | 5 | | Not Reported | | PDD and MR | | Food selectivity | | |
| Babbitt et al., 1994 (case study 3) | 1 | Parents | Not Reported | Not Reported |  | 1 | 5 | | Not Reported | | MR; cortical blindness, Dandy Walker malformation, seizure disorder and TBI | | Food refusal | | |
| Bachmeyer-Lee et al., 2020 | 6 | Parents & Grandparent | 34; 36; 38; 41; 42; 63 | HS 2; Bachelors 4 |  | 3 | 2, 3, 4 | | Not Reported | | ARFID; TD; FTT and Dysphagia and tube; DD | | Varied | | |
| Begotka et al., 2018 | 39 | Parents | Not Reported | Not Reported |  | 39 | 1-17  (mean 5.3) | | White, African American, Hispanic, Asian | | Structural Abnormalities, Neuro-Developmental Conditions, Genetics, Cardio-Resiratory Problems, Gastrointestinal, Allergy/Immune Disorder, Endocrine, Behavioral Issues, Child Internalizing Problems, Child Externalizing Problems, Sensory, Oral Feeding Skill Problem, Swallow Safety Concern | | Varied | | |
| Binnendyk & Lucyshyn, 2009 | 1 | Parent | Not Reported | Not Reported |  | 1 | 6 | | Canadian w/Middle Eastern descent | | ASD | | Food selectivity | | |
| Bloomfield et al., 2019 | 2 | BCBA^®^ & Parent | Not Reported | Not Reported |  | 1 | 8 | | White/Non-Hispanic | | TD, ARFID | | Food avoidant and restrictive | | |
| Bloomfield et al., 2021 | 2 | Staff & Parent | Not Reported | Not Reported |  | 1 | 5 | | White | | ARFID, ASD | | Food selectivity | | |
| Caldwell et al., 2020 | 20 | Parents | 27-50 (mean 34) | Vocational Degree 1; Bachelors 10; Graduate Degree 9 |  | 20 | 15: 18-35 m  5: 36-60 m | | White/Non-Hispanic | | Sensory food aversion | | Food selectivity | | |
| Cho & Sonoyama, 2020 | 4 | Parents & Teachers | Not Reported | Not Reported |  | 1 | 7 | | Brazilian | | ASD | | Food selectivity | | |
| Clark et al., 2020 | 3 | Parents | 33; 35; 38 | Bachelors |  | 3 | 3, 4, 6 | | Not Reported | | ASD | | Food selectivity | | |
| Cosbey & Muldoon, 2017 | 5 | Parents & Grandparent | Not Reported | Not Reported |  | 3 | 6, 7, 8 | | White/Hispanic 2, White 1, African American 2 | | ASD | | Food selectivity | | |
| Dadds et al., 1984 | 7 | Parents | Not Reported | Not Reported |  | 4 | 2-4 | | Not Reported | | Not reported | | Not reported | | |
| Dahlsgaard & Bodie, 2019 | 21 | Parents | Not Reported | Not Reported |  | 21 | 4-12  (mean 7.66) | | White | | ARFID (all); 8 Anxiety; 3 OCD or tic; 3 ADHD or behavior disorder | | Food selectivity | | |
| Dovey & Martin, 2012 | 17 | Parents | 23-29 (mean 33.25) | High School 16; Higher Ed. 1 |  | 17 | 28-82 m (mean 50.7) | | Not Reported | | TD | | Food selectivity | | |
| Fraser et al., 2004 | 44 | Parents | Not Reported | Mixed of High School, Higher Ed., & Not Reported |  | 75 | 2-10 | | Australian, New Zealand, European, Filipinos | | Not reported | | Not reported | | |
| Gentry & Luiselli, 2008 | 1 | Parent | Not Reported | Not Reported |  | 1 | 4 | | Not Reported | | PDD | | Food selectivity | | |
| Gutentag & Hammer, 2000 | 1 | Parent | Not Reported | Not Reported |  | 1 | 3 | | Not Reported | | DD, cardiac and pulmonary, respiratory, GI problems | | Food Refusal | | |
| Johnson et al., 2015 | 18 | Parents | Mean 37 | HS 4; Some college 6; Bachelors 6; Post Graduate 2 |  | 14 | 2-7 (mean 4.86) | | White 12; Multi ethnic 2 | | ASD | | Food selectivity | | |
| Johnson et al., 2019 | 37 | Parents | Mean 37 | HS 1; Some college 6; Bachelors 9; Post Graduate 5 |  | 37 | 2-11 (mean 5.1) | | White/Non-Hispanic 34; Black 1; Asian/Multiracial 6; Hispanic 8; Other 1 | | ASD | | Food selectivity | | |
| Kahng et al., 2001 | 2 | Parent & Grandparent | Not Reported | Not Reported |  | 1 | 5 | | Not Reported | | MR, FTT | | Food refusal | | |
| King et al., 2022 | 1 | Parent | Not Reported | Not Reported |  | 1 | 17 | | Latino | | ASD, OCD, ARFID | | Food selectivity | | |
| Linscheid et al., 1978 | 2 | Parents | Not Reported | Not Reported |  | 2 | 2, 4 | | Not Reported | | DD, physical handicap, MR spina bifida; cleft palate, systolic heart murmur, bilateral inguinal hernia, bilateral atresia of external auditory canal, impaired hearing, blind right eye, facial anomalies characteristic of Treacher-Collins syndrome, abnormal length, weight & head circumference | | Food selectivity | | |
| Mann et al., 2023 | 2 | Parents | Not Reported | Not Reported |  | 1 | 13 m | | Chinese | | Faltering growth, gastroesophageal reflux | | Food Selectivity | | |
| McCartney et al., 2005 | 4 | Parent | Not Reported | Not Reported |  | 4 | 1.5, 5, 5, 7 | | Not Reported | | 2 ASD, MR; 1 ASD; 1 TD | | Varied | | |
| Mueller et al., 2003 | 9 | Parents & Grandparent | Not Reported | Not Reported |  | 5 | Not reported | | Not Reported | | Not reported | | Severe feeding problems | | |
| Najdowski et al., 2010 | 3 | Parent | 36; 39; 43 | Bachelors 2; Post Graduate 1 |  | 3 | 2, 4, 4 | | Not Reported | | ASD; ASD; TD | | Food selectivity | | |
| O'Reilly & Lancioni, 2001 | 1 | Parent | Not Reported | Not Reported |  | 1 | 4 | | Not Reported | | Williams syndrome hypercalcemia | | Food selectivity | | |
| Pangborn et al., 2013 | 4 | Parents & Grandparent | 34; 36; 53; Not Reported | Not Reported |  | 2 | 2, 2 | | Not Reported | | FTT/GER/constipation; severe emesis, FTT | | Food refusal | | |
| Patel et al., 2023 | 5 | Parents | Not Reported | Some college |  | 5 | 2, 2, 4, 4, 5 | | White 2; Asian 3 | | DD/GER; DD/GER; ASD/constipation/caudal agenesis; TD/FTT; TD/GER | | Food refusal | | |
| Penrod et al., 2010 | 3 | Parents | 30; 35; 36 | HS 1; Bachelors 2 |  | 3 | 3, 4, 4 | | White | | 1 PDD; 2 ASD | | Food selectivity | | |
| Pizzo et al., 2009 | Not Reported | Parents | Not Reported | Not Reported |  | 3 | 4, 5, 9 | | Not Reported | | GER; ADHD; ASD | | Food selectivity | | |
| Seiverling et al., 2012 | 3 | Parents | 33; 40; 41 | Not Reported |  | 3 | 4, 5, 8 | | Not Reported | | ASD | | Food selectivity | | |
| Seiverling et al., 2018 | 3 | Parents | Not Reported | Not Reported |  | 2 | 5, 6 | | Not Reported | | ASD; ASD, asthma, pica, and Systemic Scleroderma | | Varied | | |
| Sharp et al., 2014 | 19 | Parents | Not Reported | Not Reported |  | 19 | 3-8 (mean 5.9) | | Not Reported | | ASD | | Food selectivity | | |
| Sharp et al., 2016 | Not Reported | Parents | Not Reported | Not Reported |  | 20 | 1-6 (mean 3.5) | | Not Reported | | 12 GER; 2 Allergy; 6 FTT; 9 Heart Problems; 5 Bronchopulmonary dysplasia; 7 ASD | | Food refusal | | |
| Sharp et al., 2019 | 38 | Parents | Mean 59.1 | Not Reported |  | 38 | 3-7 (mean 4.9) | | Not Reported | | ASD | | Food selectivity | | |
| Tanner et al., 2023 | 124 | Parents | Not Reported | HS 1; Some college 6; Postsecondary nondegree 2; Associates 9; bachelor’s 35; master’s 16; Doctoral 9 |  | 124 | 1.5-6 (mean 3.9) | | Majority White/Non-Hispanic | | Not reported | | Food selectivity | | |
| Tarbox et al., 2010 | 1 | Mother | Not Reported | Not Reported |  | 1 | 3 | | Not Reported | | ASD | | Food selectivity | | |
| S. Taylor et al., 2019 | Not Reported | Caregivers | Not Reported | Not Reported |  | 9 | 3-14 (mean 7.9) | | New Zealand Māori2; New Zealand European 2; Asian 3; Pacific 2; European 2 | | 5 FTT; 4 ASD; 5 DD | | Food refusal | | |
| T. Taylor, 2020 | 6 | BCBA^®^, Staff, Parents | Not Reported | Not Reported |  | 2 | 5, 4 | | Asian; White | | ASD | | Varied | | |
| T. Taylor, 2021 | 3 | Parent & Staff | Not Reported | Not Reported |  | 1 | 5 | | Asian/Australian | | ASD, ARFID | | Food selectivity | | |
| T. Taylor & Haberlin, 2020 | 4 | Parents & Staff | Not Reported | Not Reported |  | 2 | 2, 2 | | Asian/Australian; South American/Australian | | TD | | Food selectivity | | |
| Taylor & Taylor, 2024 | 2 | Parents | Not Reported | Not Reported |  | 1 | 3.5 | | Not Reported | | ASD, global developmental delay, pediatric feeding disorder, underweight | | Food selectivity | | |
| Tereshko et al., 2023 | 2 | Parents | Not Reported | Not Reported |  | 1 | 7 | | White | | ASD, anxiety | | Food selectivity | | |
| Turner et al., 1994 | 20 | Parents | Mothers’- mean 31.6 Fathers’ mean 35.7 | Varied: Junior, Senior, Tertiary Trade |  | 20 | mean 36.7 m and 28.6 m across groups | | Not Reported | | 2 g-tube feeding, 8 GER, 4 Reflux, 5 had other organic problems (bowel blockage, diarrhea) | | Varied | | |
| Werle et al., 1993 | 3 | Parents | Not Reported | Not Reported |  | 3 | 21m, 3, 5 | | Not Reported | | 2 DD; 1 TD | | Varied | | |

**Table 2**

*Experimental Design and outcome measures*

| Article | Training Components | Intervention Trained | Dependent Variable(s) | Experimental Design | Fidelity Experimental Control | Trainee Outcome | Feeding Outcome |
| --- | --- | --- | --- | --- | --- | --- | --- |
| Aclan & Taylor, 2017 | Written Instruction, Q&A, Feedback | DRA, NRS | Consumption, IMB, Fidelity | Multiple Baseline | Yes | Improved | Improved |
| Alaimo et al., 2018 | Written & Verbal Instruction, Q&A, Modeling, Rehearsal, Feedback | DRA, NRS | Consumption, IMB, Fidelity | Multiple Baseline | Yes | Improved | Improved |
| Alaimo et al., 2023 | Written & Verbal Instructions, Modeling, Feedback, Rehearsal via telehealth | EE, Limited hold | Consumption, IMB, Fidelity | Multiple Baseline | Yes | Improved | Improved |
| Anderson & McMillian, 2001 | Written & Verbal Instruction, Modeling, Videotaped reviews, Feedback | DRA, EE | Consumption, IMB, Fidelity | Reversal | No | Improved | Improved |
| Babbitt et al., 1994 (Case Study 3) | Observation, Role Playing (doll, confederate, child) | Generalized compliance training (high probability/low probability sequence) | Consumption, Fidelity | Multiple Baseline | No | Improved | Improved |
| Bachmeyer-Lee et al., 2020 | Written Instruction, Feedback | EE, AE, DRA | Consumption, IMB | Multiple Baseline | N/A | Improved | Improved |
| \| Begotka et al., 2018 \| \| --- \| | Modeling, Fading Parent into Session, Earpiece Coaching | Behavioral strategies (Differential reinforcement, Visual contingency, antecedent prompt, EE) | Feeding Questionnaires | Pre/Post Group | N/A | Not Reported | Improved |
| Binnendyk & Lucyshyn, 2009 | Modeling, Coaching, Feedback, Self-monitoring, Role Playing | Mass trials, Stimulus fading, Shaping, Contingent Reinforcement, EE | Consumption, Fidelity | Multiple Probe | Yes | Improved | Improved |
| Bloomfield et al., 2019 | Verbal Instruction, Modeling, Feedback | Stepwise changing contingency for reinforcement, guided compliance | Consumption, IMB, Fidelity | Changing Criterion | No | Improved | Improved |
| Bloomfield et al., 2021 | Written & Verbal Instruction, Rehearsal, Modeling, Feedback | DRA, Demand fading, least to most prompting | Consumption, Fidelity | Changing Criterion | No | Variable | Improved |
| Caldwell et al., 2020 | Shaping, Coaching, Feedback | Antecedent Strategies, Reinforcement | IMB, Fidelity | Repeated Measures | No | Not Reported | Improved |
| Cho & Sonoyama, 2020 | Instruction, Q&A, Feedback | Fading, Simultaneous presentation | Consumption, IMB | Reversal | N/A | Improved | Improved |
| Clark et al., 2020 | Written Instruction, Video Modeling, Coaching, Feedback | Prompting, DRA | Consumption, Fidelity | Multiple Baseline | Yes | Improved | Improved |
| Cosbey & Muldoon, 2017 | Coaching, Observation, Verbal Instruction, Visual Supports, Feedback | Antecedent, Shaping, Reinforcement | Consumption, IMB | Multiple Baseline | N/A | Improved | Improved |
| Dadds et al., 1984 | Mealtime Management Training- Modeling, Instruction, Feedback | DRA, Behavior correction routines, AE, Timeout | Consumption, IMB, Fidelity | Multiple Baseline | No | Improved | Variable |
| Dahlsgaard & Bodie, 2019 | Verbal Instruction, Coaching, Role Play, Q&A | Picky Eaters Clinic Intervention (DRA, Contingency management, habituation, exposure, fading) | Child Feeding Behaviors, Fidelity | Pre/Post Group | No | Improved | Improved |
| Dovey & Martin, 2012 | Discussion | Contingent reinforcement | Child Feeding Behaviors | Pre/Post Group | N/A | Not Reported | Improved |
| Fraser et al., 2004 | Verbal Instruction, Role Modeling, Reinforcement, Stimulus Control Techniques | Fun not Fuss with Food Program | Consumption, IMB | Quasi-Experimental Time Series | N/A | Not Reported | Improved |
| Gentry & Luiselli, 2008 | Verbal Instruction, Rehearsal, Feedback | Visual Cue, Sequential, Reinforcement | Consumption | Changing Criterion | N/A | Not Reported | Improved |
| Gutentag & Hammer, 2000 | Modeling | DRA, Instructional Control, Timeout | Consumption; IMB | Multiple Baseline | N/A | Improved | Improved |
| Johnson et al., 2015 | Instruction, Modeling, Role Playing | A manualized behavioral parent training program | Child feeding behaviors, IMB, Caregiver stress, Fidelity, Consumption | Pre/Post Group | No | Improved | Improved |
| Johnson et al., 2019 | Instruction, Modeling, Role Playing | A manualized behavioral parent training program | Child feeding behaviors, Caregiver stress, Fidelity, Consumption | Parallel Group Trial | No | Improved | Improved |
| Kahng et al 2001 | Instructions, Observation, Feedback | Response Cost, DRA | Consumption, IMB, Fidelity | Reversal | No | Improved | Improved |
| King et al., 2022 | Written & Verbal Instruction, Modeling, Q&A, Feedback | DRA, Contingency Management | Consumption, IMB, Fidelity, Acceptability | Multiple Baseline | No | Improved | Improved |
| Linscheid et al., 1978 | Modeling, Feedback | DRA; Sequential Presentation, Timeout | Consumption, IMB, Fidelity | Reversal | No | Not Reported | Improved |
| Mann et al., 2023 | Written & Verbal Instruction, Modeling, Rehearsal, Feedback | DRA, Volume Fading | Consumption, IMB | Multiple Probe | N/A | Improved | Improved |
| McCartney et al., 2005 | Modeling, Verbal Instruction, Observation, Feedback | EE, Differential Reinforcement | Consumption, IMB | Changing Criterion with within Subject Replication | N/A | Improved | Improved |
| Mueller et al., 2003 | Written & Verbal Instruction, Modeling, Rehearsal, Feedback | DRA, NCR, NRS | Consumption, IMB, Fidelity | Multiple Baseline | Yes | Improved | Improved |
| Najdowski et al., 2010 | Written & Verbal Instructions, Role play, Coaching | DRA, NRS, Demand Fading | Consumption, IMB, Fidelity | Multiple Baseline | No | Improved | Improved |
| O'Reilly & Lancioni, 2001 | Modeling, Rehearsal | DRA, Physical Prompting | Consumption, IMB | Multiple Baseline | N/A | Improved | Improved |
| Pangborn et al., 2013 | Written & Verbal Instructions, Q&A, Quiz, Feedback, Role play, Modeling | EE, Physical and Vocal Prompt | Consumption, IMB, Fidelity | Multiple Baseline | Yes | Improved | Improved |
| Patel et al., 2023 | Written Instruction, Modeling, Role play, Feedback | NCR, Response Cost, NRS, Representation | Consumption, Fidelity | Multiple Baseline | No | Improved | Improved |
| Penrod et al., 2010 | Written & Verbal Instructions, Role play, Coaching, Feedback | DRA, EE, Fading, Reinforcer Manipulation | Consumption, IMB, Fidelity | Multiple Baseline | No | Improved | Improved |
| Pizzo et al., 2009 | Instructions, Rationale | Taste Exposure, DRA, Fading | Consumption, IMB | Multiple Baseline | N/A | Not Reported | Improved |
| Seiverling et al., 2012 | Verbal Instruction, Modeling, Rehearsal, Feedback | DRA, EE, NCR | Consumption, IMB, Fidelity | Multiple Baseline | No | Improved | Improved |
| Seiverling et al., 2018 | Written Instruction, Modeling, Role play | Behavioral Feeding Protocol | Consumption, IMB | Alternating Treatment | N/A | Not Reported | Improved |
| Sharp et al., 2014 | Written & Verbal Instructions | Autism MEAL plan | Indirect measures | RCT | N/A | Not Reported | Improved |
| Sharp et al., 2016 | Verbal Instruction, Feedback | Integrated eating aversion treatment (iEAT) | Consumption, IMB | RCT | N/A | Improved | Improved |
| Sharp et al., 2019 | Written Instruction, Homework, Role play, Coaching | Autism MEAL plan | Indirect measures; Consumption, Fidelity | RCT | No | Improved | Improved |
| Tanner et al., 2023 | Written Instruction, Video Models | Behavioral Feeding Guidelines | Indirect measures; Consumption, IMB | Randomized Control Trial | N/A | Improved | Variable |
| Tarbox et al., 2010 | Consultation, Feedback, Verbal Instructions, Q&A | Rules, Reinforcer manipulation, EE | Consumption | Reversal | N/A | Improved | Improved |
| S. Taylor et al., 2019 | Observation, Instruction, Role play, Feedback | Antecedent Strategies, Reinforcement, Extinction | Consumption, Fidelity | Multiple Baseline | Yes | Improved | Improved |
| T. Taylor, 2020 | Observation, Written & Verbal Instruction, Role play, Fading | DRA, DRA+NRS, Physical Prompt, Side Deposit, Response Cost | Consumption, IMB, Fidelity | Reversal | No | Improved | Improved |
| T. Taylor, 2021 | Observation, Written & Verbal Instruction, Role play, Fading | DRA, NRS, Representation, Physical Prompt | Consumption, IMB, Fidelity | Reversal | No | Improved | Improved |
| T. Taylor & Haberlin, 2020 | Instruction, Modeling, Rehearsal, Feedback | DRA, NRS, Representation | Consumption, IMB, Fidelity | Reversal | No | Improved | Improved |
| Taylor & Taylor, 2024 | Written & Verbal Instructions, Modeling, Feedback, Rehearsal, General Case Training, Video Models via Telehealth | Differential attention, Reinforcer Manipulation, EE | Consumption | Multiple Baseline | N/A | Improved | Improved |
| Tereshko et al., 2023 | Verbal Instruction, Modeling via telehealth and in person | DRA, Visuals, Choice | Consumption, IMB | Multiple Baseline & Modified Changing Criterion | N/A | Improved | Improved |
| Turner et al., 1994 | Written & Verbal Instructions, Modeling, Feedback, Rehearsal | DRA, Timeout, Shaping, Prompting, Prompt Fading | Consumption, IMB, Fidelity | Randomized Group Comparison | No | Improved | Improved |
| Werle et al., 1993 | Written & Verbal Instruction, Discussion, Role play, Rehearsal, Feedback | DRA, Prompting, Timeout | Consumption, IMB, Fidelity | Multiple Baseline | Yes | Improved | Improved |

**Table 3**

*Data across measures of quality*

| Article | Generalization | Maintenance | Social Validity | Social Validity Assessment | Social Validity Outcome |
| --- | --- | --- | --- | --- | --- |
| Aclan & Taylor, 2017 | Intervention | 6 days; 3 & 6 weeks | No | NA | N/A |
| Alaimo et al., 2018 | Intervention | 1 & 3 weeks | Parents | Likert Scale Questionnaire | Intervention- positive  Training- positive |
| Alaimo et al., 2023 | No | 2-3 weeks | Parent |  | Intervention- positive  Training- positive |
| Anderson & McMillian, 2001 | No | No | No | N/A | N/A |
| Babbitt et al., 1994 (Case Study 3) | Intervention | No | No | N/A | N/A |
| Bachmeyer-Lee et al., 2020 | No | 1 & 2 weeks, 1 month | Parents | Questionnaire | Training- positive |
| \| Begotka et al., 2018 \| \| --- \| | No | No | No | N/A | N/A |
| Binnendyk & Lucyshyn, 2009 | Both | 1, 5, & 6 weeks, 26 months | Parents | Likert Scale Questionnaire | Intervention- positive |
| Bloomfield et al., 2019 | No | 1 & 4 months | Parents | FFTAM; BIRS | Intervention- positive |
| Bloomfield et al., 2021 | No | No | Parent | Informal | Intervention- positive |
| Caldwell et al., 2020 | No | No | No | N/A | N/A |
| Cho & Sonoyama, 2020 | Intervention | 1 & 3 months | Parents & Teacher | TARF-R | Intervention- positive |
| Clark et al., 2020 | No | No | Parents | Likert Scale Questionnaire | Training- positive |
| Cosbey & Muldoon, 2017 | No | 6 months | Parents | BAMBI; BPFAS | Intervention- positive |
| Dadds et al., 1984 | No | 3 months | No | N/A | N/A |
| Dahlsgaard & Bodie, 2019 | No | 3 months | Parents | Likert Scale Questionnaire | Intervention- positive |
| Dovey & Martin, 2012 | No | No | No | N/A | N/A |
| Fraser et al., 2004 | No | 1 month | No | N/A | N/A |
| Gentry & Luiselli, 2008 | No | No | No | N/A | N/A |
| Gutentag & Hammer, 2000 | No | No | No | N/A | N/A |
| Johnson et al., 2015 | No | Yes- duration not specified | Parents | PSQ | Training- positive |
| Johnson et al., 2019 | No | No | Parents | PSQ | Intervention- positive |
| Kahng et al 2001 | No | No | No | N/A | N/A |
| King et al., 2022 | Intervention | 1.5 months | Parents | FF-TAM | Intervention- positive  Training- neutral |
| Linscheid et al., 1978 | Intervention | 4 days, 2 weeks, 2 & 3 months | No | N/A | N/A |
| Mann et al., 2023 | Intervention | 1, 3, 6, 12 months | Parent | Likert Scale Questionnaire | Intervention- positive  Training- positive |
| McCartney et al., 2005 | Intervention | 4 weeks, 1 year | Parents | Likert Scale Questionnaire | Intervention- positive  Training- neutral |
| Mueller et al., 2003 | Intervention | 6 days to 3 months | No | N/A | N/A |
| Najdowski et al., 2010 | Intervention | 2, 4, 6, 8, & 12 weeks | Parents | Modified Published Questionnaire | Intervention- positive |
| O'Reilly & Lancioni, 2001 | No | 1, 2, & 3 months | No | N/A | N/A |
| Pangborn et al., 2013 | No | No | Parents | Likert Scale Questionnaire | Training- positive |
| Patel et al., 2023 | No | 1 year | Parents | Likert Scale Questionnaire | Intervention- positive  Training- positive |
| Penrod et al., 2010 | Intervention | 2, 4, 6, & 12 weeks | Parents | Modified Published Questionnaire | Intervention- positive |
| Pizzo et al., 2009 | Intervention | 4 weeks | No | N/A | N/A |
| Seiverling et al., 2012 | Intervention | 3 months | Parents | Likert Scale Questionnaire | Intervention- positive  Training- positive |
| Seiverling et al., 2018 | Intervention | 1 & 2 months | No | N/A | N/A |
| Sharp et al., 2014 | No | No | Parents | Likert Scale Questionnaire | Intervention- neutral  Training- positive |
| Sharp et al., 2016 | Intervention | 36 days | Parent | Likert Scale Questionnaire | Intervention- positive |
| Sharp et al., 2019 | No | 4 weeks | Parents | Likert Scale Questionnaire | Intervention- positive |
| Tanner et al., 2023 | No | No | Parents | Questionnaire | Training- Positive |
| Tarbox et al., 2010 | No | 1, 2, 4, 9 weeks | Parent | Informal | Intervention- Positive |
| S. Taylor et al., 2019 | No | 1, 3, 6, & 12 months | Parents | PIP | Intervention- positive |
| T. Taylor, 2020 | Intervention | 2 weeks, 1, 3, & 6 months, 1 & 3 years | No | N/A | N/A |
| T. Taylor, 2021 | Intervention | 1 month | Parents | Likert Scale Questionnaire | Intervention- positive |
| T. Taylor & Haberlin, 2020 | Intervention | 2 weeks, 1.5 & 3 years | Parents | Likert Scale Questionnaire | Intervention- positive |
| Taylor & Taylor, 2024 | Intervention | 6, 12 months | Parent | Likert Scale Questionnaire | Intervention- positive |
| Tereshko et al., 2023 | Intervention | 1, 2, 4 weeks | Parent | Likert Scale Questionnaire | Intervention- positive  Training- positive |
| Turner et al., 1994 | No | 3-4 months | Parents | Questionnaire | Training- positive |
| Werle et al., 1993 | No | No | No | N/A | N/A |
